# PTX3 Predicts Myocardial Damage and Fibrosis in Duchenne Muscular Dystrophy

**DOI:** 10.3389/fphys.2020.00403

**Published:** 2020-05-19

**Authors:** Andrea Farini, Chiara Villa, Dario Di Silvestre, Pamela Bella, Luana Tripodi, Rossana Rossi, Clementina Sitzia, Stefano Gatti, Pierluigi Mauri, Yvan Torrente

**Affiliations:** ^1^Stem Cell Laboratory, Department of Pathophysiology and Transplantation, Università degli Studi di Milano, Unit of Neurology, Fondazione IRCCS Ca’ Granda Ospedale Maggiore Policlinico, Centro Dino Ferrari, Milan, Italy; ^2^Institute of Technologies in Biomedicine, National Research Council (ITB-CNR), Milan, Italy; ^3^Residency Program in Clinical Pathology and Clinical Biochemistry, Università degli Studi di Milano, Milan, Italy; ^4^Center for Surgical Research, Fondazione IRCCS Cà Granda, Ospedale Maggiore Policlinico, Milan, Italy

**Keywords:** Duchenne muscular dystrophy (DMD), muscular dystrophy, cardiomyopathy, pentraxin 3 (PTX3), alarmins

## Abstract

Pentraxin 3 (PTX3) is a main component of the innate immune system by inducing complement pathway activation, acting as an inflammatory mediator, coordinating the functions of macrophages/dendritic cells and promoting apoptosis/necrosis. Additionally, it has been found in fibrotic regions co-localizing with collagen. In this work, we wanted to investigate the predictive role of PTX3 in myocardial damage and fibrosis of Duchenne muscular dystrophy (DMD). DMD is an X-linked recessive disease caused by mutations of the dystrophin gene that affects muscular functions and strength and accompanying dilated cardiomyopathy. Here, we expound the correlation of PTX3 cardiac expression with age and Toll-like receptors (TLRs)/interleukin-1 receptor (IL-1R)-MyD88 inflammatory markers and its modulation by the so-called alarmins IL-33, high-mobility group box 1 (HMGB1), and S100β. These findings suggest that cardiac levels of PTX3 might have prognostic value and potential in guiding therapy for DMD cardiomyopathy.

## Introduction

Pentraxins (PTXs) are a superfamily of proteins containing the highly conserved C-terminal PTX domain. According to the primary structure of the promoter, they are divided into two distinct groups: short and long (Deban et al., 2011). Among the longer subfamily, the Pentraxin 3 (PTX3) is an inflammatory mediator, mainly produced during the first phase of the inflammatory processes by phagocytes, neutrophils, fibroblasts, endothelial cells, following the secretion of inflammatory cytokines (Doni et al., 2008). PTX3 is a fundamental component of humoral innate immunity and – in synergy with other proteins as the PTX C-reactive protein (CRP) and serum amyloid P-component (SAP) – mediates the innate resistance to pathogens, allows the activation of complement pathway, coordinates the functions of macrophages/dendritic cells (DCs), and fosters apoptosis/necrosis (Garlanda et al., 2005; Liu et al., 2014). PTX3 plays a role in vessel repair and remodeling (Presta et al., 2007; Deban et al., 2010), and it has been documented synthesized by endothelial and smooth muscle cells as well as granulocytes at sites of active vasculitis (Fazzini et al., 2001; Presta et al., 2007; Castellano et al., 2010; Cieslik and Hrycek, 2015). Moreover, PTX3 participates in the regulation of inflammation as well as in extracellular matrix formation promoting fibrocyte differentiation (Pilling et al., 2015). However, in acute myocardial infarction as well as in myocarditis, increased PTX3 expression of both macrophages and endothelial cells was found (Nebuloni et al., 2011). In the heart, PTX3 can be induced by MyD88, which is a canonical adaptor for inflammatory signaling pathways downstream of members of the Toll-like receptor (TLR) and interleukin-1 (IL-1) receptor families. The TLRs/IL-1R-MyD88 signaling can lead to distinct outputs depending on the context: pro-inflammatory by the activation of transcription factor NF-κB (Kunes et al., 2012) or anti-inflammatory *via* type I interferon production or complement binding (Salio et al., 2008). Inflammatory signals might also participate in PTX3 expression by the catalytic activity of the induced form of the proteasome [the immunoproteasome (IP)] together with the mitogen-activated protein (MAP)-kinases p38 and extracellular signal-regulated kinase (ERK)1/2 (Paeschke et al., 2016). According to its dual role, the cardioprotective function of PTX3 has been demonstrated in acute myocardial infarction (Salio et al., 2008; Casula et al., 2017). Interestingly, Liu et al. (2018) demonstrated that PTX3 was able to modulate the expression of several cardiac genes and enhanced the transformation of mouse embryonic stem cells into cardiomyocytes. Among inflammatory signals, the IL-33 and its receptor sST2 have been associated with the overexpression of PTX3 following cardiac infarction (Ristagno et al., 2015). IL-33 is a member of the IL-1 family of cytokines produced by primarily non-hematopoietic cells in response to mechanical stress and injury. IL-1/IL-33 and other proteins as HMGB1, S100β are the so-called alarmins that are released by both resident immune cells and necrotic cells that underwent damage. These alarmins are then recognized by specific receptors [e.g., the receptor for advanced glycation end-products (RAGE) and TLRs] of various immune cells that initiate inflammatory and repair responses.

More interestingly, the IL-33/ST2 system emerged as a novel fibroblast–cardiomyocyte communication system that regulates the accumulation of anti-inflammatory T regulatory lymphocytes (Tregs) and was proposed as a biomarker for cardiomyopathy in Duchenne muscular dystrophy (DMD) (Kuswanto et al., 2016). DMD cardiomyopathy is the major cause of mortality for DMD patients, and it is characterized by unresolved cardiac inflammation and fibrosis. Recently, Frohlich et al. (2016) confirmed high expression of PTX3 in DMD dystrophic animal models. Taking into account the critical role of PTX3 in the inflammatory/fibrotic pathways and the absence of predictor markers of cardiomyopathy in DMD patients, we argued to investigate the role of PTX3 in myocardial damage and fibrosis of the *mdx* mouse model for DMD. Dystrophic cardiac expression of PTX3 correlated positively with age and inflammatory/fibrotic pathways, suggesting that cardiac levels of PTX3 have prognostic value and potential in guiding therapy for cardiomyopathy of DMD.

## Materials and Methods

### Animal Statement

All procedures involving living animals were performed in accordance with Italian law (D.L.vo 116/92 and subsequent additions), which conforms to the European Union guidelines. The use of animals in this study was authorized by the National Ministry of Health (protocol number 10/13–2014/2015). Ten weeks (10w), 3 months (3m), 5m, and 7m C57Bl and 11 days (11dy), 10w, 3m, 9m, 14m, and 18m mdx (*C57BL6/*10ScSn-DMD*mdx*/J) mice were provided by Charles River. All animals were housed in a controlled ambient environment (12 h light/dark cycle) at a temperature between 21 and 23°C. Cage population was limited to a maximum of four animals each to ensure the health and welfare of animals. The mice had free access to clean water and food. Systemic intraperitoneal injection of the IP inhibitor ONX-0914 (Clini Sciences, 6 mg/kg) was performed in 10w and 9m mdx mice for 5 weeks (two injections/week, *n* = 10). Untreated aged-matched mdx mice were used as controls. After 1 month of treatment, mice were deeply anesthetized with 2% avertin (0.015 ml/kg), then sacrificed by cervical dislocation.

### RT-qPCR Experiments

Total RNA was extracted from cardiac tissues obtained from 11dy, 10w, 3m, 9m, and 18m mdx mice. cDNA was generated using the Reverse Transcriptase Kit (Thermo Fisher Scientific) followed by the SYBR-Green reaction to quantify the expression of the genes in Table 1. All the cDNA samples were tested in duplicate, and the threshold cycles (Ct) of target genes were normalized against a housekeeping gene, the glyceraldehyde 3-phosphate dehydrogenase (GAPDH). Relative transcript levels were calculated from the Ct values as X = 2^–Δ^ ^Δ^ ^ct^ where X is the fold difference in the amount of target gene versus GAPDH and ΔCt = Ct_*target*_−Ct_*GAPDH*_.

**TABLE 1:** Sequence of primers used in RT-qPCR.

	Forward (5′→3′)	Reverse (5′→3′)
C1s	tgaaggaagagggaaagacaag	gattttggaggtaaagggcagt
C1r	acttccgctacatcaccacaa	ctctccttcctcttcattcttcc
C3	acaaactcacacagagcaaga	atccatgaagacaccagcatag
C5	cagcaaggaggagtcaacat	tccacaagagcccgtaaatc
FH	tctcaggctcgtggtcagaa	ccagggcggcatttgtag
C4	tctcacaaacccctcgacat	agcatcctggaacacctgaa

### Network-Based Prediction of Protein Interactions

A protein–protein interaction (PPI) network (30 nodes and 70 edges) was built by matching differentially expressed proteins and *Mus musculus* PPI data retrieved from STRING (Szklarczyk et al., 2019)^1^; only Experimental and Database annotated interactions (Score > 0.15) were considered (Doncheva et al., 2019). Protein expression data were further processed by Pearson’s correlation (Score > | 0.6|, *p* < 0.05), and significant correlations were visualized as a network (27 nodes and 99 edges) using Cytoscape software (Vella et al., 2017). Reconstructed network was analyzed at the topological level, and Cytoscape’s plugin CentiScaPe was used to calculate centrality indices of each node (Scardoni et al., 2009); specifically, nodes with Betweenness, Bridging, and Degree above the network average were retained and considered hubs as previously reported (Sereni et al., 2019).

### Western Blot Analysis

Total proteins from skeletal muscles and cardiac biopsies isolated from normal and dystrophic mice were extracted as in Farini et al. (2019). Samples were resolved on polyacrylamide gels (ranging from 10% to 14%) and transferred to nitrocellulose membranes (Bio-Rad Laboratories). Filters were incubated overnight with the following antibodies: vinculin (1:600, MA5-11690, Invitrogen); PTX3 (C-10 1:600, sc-373951, Santa Cruz Biotechnology); phospho-p38 (Thr180) (1:500, E-AB-20949, Elabscience); p38 (1:500, E-AB-32460, Elabscience); ERK1/2 (1:500, E-AB-31374, Elabscience); phospho-ERK1/2 (Thr202) (1:500, E-AB-20868, Elabscience); PSMB5 (1:500, ab3330, Abcam); PSMB8 (1:500, Proteasome 20S LMP7, ab3329, Abcam); PSMB9 [1:500, Proteasome 20S LMP2 (EPR13785) ab184172, Abcam]; RAGE (1:500, NBP2-03950, Novusbio); S-100β chain (C-3) (1:500, sc-393919, Santa Cruz Biotechnology); monocyte chemoattractant protein (MCP)-1 (ECE.2) (1:500, sc-52701, Santa Cruz Biotechnology); Foxp3 (FJK-16s) (1:500, 14-5773-82, eBioscience); IL-33 (1:500, AF3626, R&D Systems); collagen VI (1:500, ab6588, Abcam); phospho-SMAD2/3 (Thr8) (1:500, E-AB-21040, Elabscience); TLR9 (26C593) (1:500, sc-52966, Santa Cruz Biotechnology); matrix metalloproteinase (MMP)-9 (E-11) (1:500, sc-393859, Santa Cruz Biotechnology); TLR4 (25) (1:500, sc-293072, Santa Cruz Biotechnology); TRAF6 (D-10) (1:500, sc-8409, Santa Cruz Biotechnology); SMAD3 (1:500, E-AB-32921, Elabscience); TLR2 (1:500, orb229137, Biorbyt); TLR5 (19D759.2) (1:500, sc-57461, Santa Cruz Biotechnology); NF-κB p65 (A-12) (1:500, sc-514451, Santa Cruz Biotechnology); RelB (D-4) (1:500, sc-48366, Santa Cruz Biotechnology); MYD88 (1:500, 23230-1-AP, Proteintech); transforming growth factor (TGF)β1 (1:500, E-AB-33090, Elabscience); IL-6 (10E5) (1:500, sc-57315, Santa Cruz Biotechnology); tumor necrosis factor (TNF)α (1:500, E-AB-40015, Elabscience); poly(ADP-ribose) polymerase (PPAR)γ (1:600, ab-59256, Abcam); autophagy-related (ATG)7 (1:600, SAB4200304, Sigma Aldrich); p62 (1:600, P0067, Sigma Aldrich); LC3B (1:500, L7543, Sigma Aldrich); HMGB1 (HAP46.5) (1:600, sc-56698, Santa Cruz Biotechnology); actin (1:600, A2066, Sigma Aldrich); GAPDH (0411) (1:600, sc-47724, Santa Cruz Biotechnology). Membranes were incubated with primary antibodies ON at 4°C, followed by washing, detection with horseradish peroxidase (HRP)-conjugated secondary antibodies (DakoCytomation, United States), and developed by enhanced chemiluminescence (ECL) (Amersham Biosciences, United States). Bands were visualized using an Odyssey Infrared Imaging System (Li-COR Biosciences, United States). Densitometric analysis was performed using ImageJ software^2^.

### Immunofluorescence and Immunohistochemistry Analysis

Cardiac biopsies were collected from 10w C57Bl and 10w, 9m, and 18m mdx mice, frozen in liquid nitrogen-cooled isopentane, and then sectioned on a cryostat (LEICA CM 1850). Serial sections (8 μm thick) were stained with Azan Mallory. Densitometric analyses and manual or automatic counting (Threshold color Plug-in) were performed using ImageJ software^2^ in 20 sections/muscle.

For immunofluorescence staining, sections were fixed in 4% paraformaldehyde (PFA) methanol-free (28908, Thermo Fisher Scientific) and permeabilized with phosphate-buffered saline (PBS) 1 × + 0.1% Triton X-100 (T9284, Sigma Aldrich) for 20 min at room temperature (RT). Sections were then incubated with PBS 1× + 10% donkey serum (blocking solution) for 1 h at RT. Primary antibodies anti-PTX3 (C-10, sc-373951, Santa Cruz Biotechnology), anti-CD31 (MEC 13.3, 5550274, BD Pharmingen), and anti-NG2 (AB5320, Merck Millipore) were diluted 1:100 in blocking solution and added to slides overnight at 4°C. Alexa Fluor-conjugated secondary antibodies against mouse (A32766, Thermo Fisher Scientific, for PTX3), rat (A21209, Thermo Fisher Scientific, for CD31), and rabbit (A31573, Thermo Fisher Scientific, for NG2) were diluted 1:200 in PBS and applied onto slides for 1 h at RT. Phycoerythrin (PE)-conjugated CD206 antibody (1:50, 141705, BioLegend) and fluorescein isothiocyanate (FITC)-conjugated α-smooth muscle actin (1:150, 1A4, Sigma Aldrich) were directly diluted in PBS and incubated for 2 h. Nuclei were counterstained with 4’,6-diamidino-2-phenylindole (DAPI), and slides were mounted with Fluoromount-G mounting medium (00-4958-02, Thermo Fisher Scientific). Images were captured using the epifluorescence microscopy DMi8 (Leica, Germany).

Immunohistochemistry of PTX3 and PMSB8 (1:100, ab3329, Abcam) was performed by blocking endogenous peroxidase activity in 0.3% alcoholic hydrogen peroxide for 30 min. Antigen retrieval was then performed in 0.01 M sodium citrate buffer at pH 6 for 30 min at 100°C. Sections were blocked with 5% horse and 5% fetal bovine serum for 30 min at RT and incubated overnight at 4°C with either anti-PTX3 or anti-PMSB8, diluted 1:100 in blocking solution. Cardiac tissues were then incubated with appropriate biotinylated immunoglobulin antibodies for 30 min at RT, followed by peroxidase–avidin–biotin complex (Vectastain ABC Elite kit; Vector Labs, Burlingame, CA, United States) incubation for 30 min. 3,3′-Diaminobenzidine (DAB) was used as the chromogen.

### Availability of Data

The raw data supporting the conclusions of this manuscript will be made available by the authors, without undue reservation, to any qualified researcher.

### Statistics

Data were analyzed by GraphPad Prism^TM^ and expressed as mean ± SD or mean ± SEM. To compare multiple group means, one-way ANOVA followed by Tukey’s multiple comparison test was used to determine significance (^∗^*p* < 0.05, ^∗∗^*p* < 0.01, ^∗∗∗^*p* < 0.001; ^****^p < 0.0001). To compare two groups, Student’s *t*-test was applied assuming equal variances: difference was considered significant at ^∗^*p* < 0.05. To correlate protein’s expression, linear regression and multivariate regression analyses were performed.

## Results

### Pentraxin 3 Expression in Skeletal Muscle of Mdx Mice Is Strictly Upregulated and Dependent on Age

**FIGURE 1:**
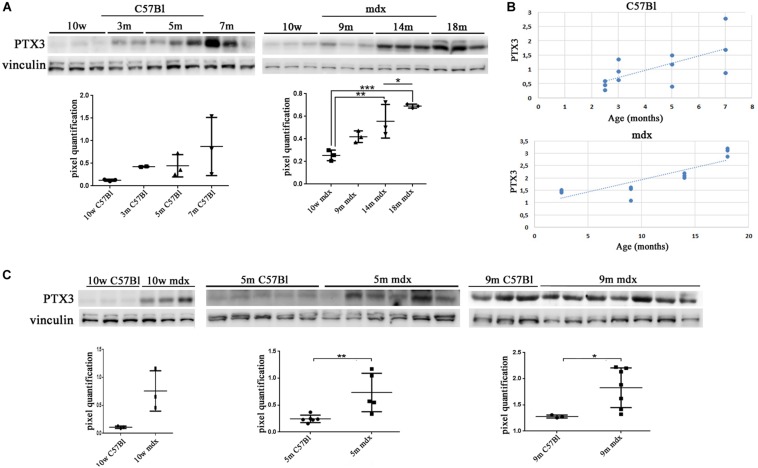
Pentraxin (PTX)3 expression in skeletal muscle biopsies of mdx mice at different ages. Representative Western blot (WB) of PTX3 in TAs of 10 weeks (10w), 3 months (3m), 5m, 7m C57Bl mice and in TAs of 10w, 9m, 14m, and 18m mdx mice. Data from densitometric analysis are expressed as PTX3/vinculin ratio in arbitrary units in the lower panels. One-way ANOVA with Tukey’s multiple comparisons test: *p < 0.05; **p < 0.01; ***p < 0.001 **(A)**. In the lateral panels, the scheme representing PTX3 expression, in C57Bl and mdx mice, according to ages **(B)**. Representative WB of PTX3 in TA of 10w, 5m, and 9m C57Bl mice and age-matched mdx mice. Data from densitometric analysis are expressed as the ratio of PTX3/vinculin in arbitrary units in the lateral panels. Student’s *t*-test: *p < 0.05; **p < 0.01 **(C)**. Each experiment was performed in triplicate wells. All values are expressed as the mean ± SD.

We firstly evaluated the expression of PTX3 in muscular biopsies of normal and dystrophic mice. In C57Bl, we determined a non-significant increase of PTX3 expression with age (Figure 1A). Conversely, we found a PTX3 downregulation in 10w C57Bl mice compared to mdx mice in the 10 weeks–18 months age range (C57Bl versus 14m mdx, *p* = 0.0044; C57Bl versus 18m mdx, *p* = 0.0005). Moreover, the dystrophic mice displayed an age-related upregulation of PTX3, particularly significant considering the older mdx (10w mdx versus 14m mdx, *p* = 0.0071; 10w mdx versus 18m mdx, *p* = 0.0005; 9m mdx versus 18m mdx, *p* = 0.0139) (Figure 1A). Statistical analysis through Pearson’s correlation coefficient also showed that PTX3 amount was dramatically dependent on the age (Pearson *r* = 0.8533; 95% confidence interval: 0.5475–0.9581 with *p* = 0.0004) (Figure 1B). We thus compared the amount of PTX3 in aged-matched muscular biopsies (10w, 5 m, 9 m), and we found a significant upregulation of PTX3 in 10w (*p* = 0.0365), 5m (*p* = 0.0089), and 9m (*p* = 0.0415) mdx related to C57Bl mice (Figure 1C).

### Pentraxin 3 Is Upregulated in Cardiac Muscles of Mdx Mice in Age-Dependent Manner

**FIGURE 2:**
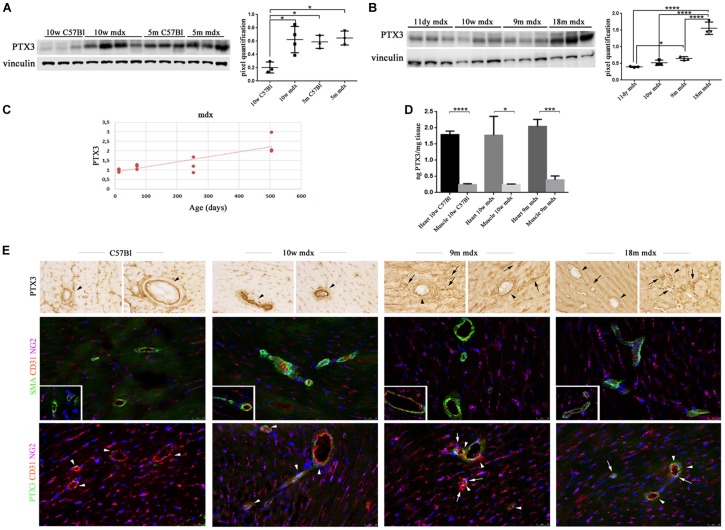
Pentraxin (PTX)3 expression in skeletal cardiac tissues of mdx mice at different ages. Representative Western blot (WB) of PTX3 in cardiac tissues of 10 weeks (10w) and 5 months (5m) C57Bl mice and age-matched mdx mice. Data from densitometric analysis are expressed as the ratio of PTX3/vinculin in arbitrary units in lateral panels. One-way ANOVA with Tukey’s multiple comparisons test: *p < 0.05 **(A)**. Representative WB of PTX3 in cardiac tissues of 11 days (11dy), 10w, 9m, and 18m mdx mice. Data from densitometric analysis are expressed as PTX3/vinculin ratio in arbitrary units in the lateral panel. One-way ANOVA with Tukey’s multiple comparisons test: *p < 0.05; ****p < 0.0001 **(B)**. The line graph represents the dependence of PTX3 expression according to the age in mdx cardiac tissues **(C)**. ELISA quantification of PTX3 in cardiac muscles of 10w C57Bl and 10w and 9m mdx mice related to skeletal muscles of age-matched mice. Student’s *t*-test: *p < 0.05; ***p < 0.001; ****p < 0.0001 **(D)**. **(E)** Representative images of hearts from 10w C57Bl and 10w, 9m, and 18m mdx mice (*n* = 5 each) showing PTX3 and vascular staining. Representative images of PTX3 immunohistochemistry staining of endothelial (arrowheads) and mural cells (pericytes) (arrows) within coronary vessel wall from C57Bl and mdx (first row). Representative images of coronary vessels expressing smooth muscle actin (SMA) (green) and CD31 (red) showing increased expression of NG2 (magenta) and altered morphology of NG2-expressing pericytes around and between cardiac vessels in 9m and 10m mdx hearts compared to C57Bl (second row). Magnification in the second row inserts indicates the presence of NG2 + pericyte processes covering C57Bl cardiac vessels, while mdx pericyte processes are spread out along the cardiac vessels. Representative images of PTX3 (green), CD31 (red), and NG2 (magenta) staining in hearts indicate PTX3-expressing endothelial CD31 + cells (arrowheads) and PTX3-expressing NG2 + pericytes (arrows) between vessels. These latter cells displayed a rounder morphology in mdx than in C57Bl hearts, and the lack of a complete covering of a large portion of cardiac vessels by NG2 + pericyte processes (third row). Nuclei were counterstained with 4′,6-diamidino-2-phenylindole (DAPI) (blue). Scale bar: 25 mm. Each experiment was performed in triplicate wells. All values are expressed as the mean ± SD.

We then aimed at determining the PTX3 role in the cardiac muscle, by assessing its amount in 10w and 5m C57Bl and mdx mouse hearts. We found a PTX3 expression increasing with ages in control mice (10w C57Bl versus 5m C57Bl, *p* = 0.0318), while both in younger and older dystrophic hearts, PTX3 was upregulated compared to 10w C57Bl (with *p* = 0.0140 and *p* = 0.0152 related to mdx 10w and mdx 5m, respectively) (Figure 2A). In line with the experiments involving skeletal muscles, we evaluated PTX3 expression in mdx mice at different ages—11dy, 10w, 9m, 18m. This time, we showed that PTX3 was only slightly upregulated in 9m related to 11dy mdx (*p* = 0.0458) (Figure 2B), but presenting a dramatic increase toward 18 months of age (with *p* < 0.0001 related to all the other mdx mice). We suggested that this condition was correlated to mdx cardiomyopathy onset, becoming evident from 8 months of age and worsening later than in skeletal muscles. Interestingly, the expression of PTX3 was age-dependent (Pearson *r* = 0.8342; 95% confidence interval: 0.4993–0.9522 with *p* = 0.0007) (Figure 2C). ELISA quantification showed that the amount of PTX3 was fivefold higher in cardiac muscles of C57Bl and 10w and 9m mdx mice related to skeletal muscles of age-matched mice (with *p* < 0.0001, *p* = 0.0106, *p* = 0.0003, respectively) (Figure 2D). We also investigated the expression of PTX3 by immunohistochemistry and immunofluorescence staining in cardiac tissues of C57Bl and mdx mice. Interestingly, we noted an increased signal for proteoglycan neural/glial2 (NG2) and an altered morphology of NG2-expressing pericytes in 9m and 18m mdx compared with 10w C57Bl and 10m mdx hearts (Figure 2E). Moreover, a lessened fluorescent PTX3 signal was found in CD31 + endothelial cells of both younger C57Bl and mdx hearts, whereas PTX3 staining become increased in CD31 + endothelial cells of 9m and 18m mdx hearts (Figure 2E). On the other hand, PTX3-expressing NG2 + pericytes between cardiac vessels with a rounder morphology were present only in 9m and 18m mdx (Figure 2E).

### Pentraxin 3 in Dystrophic Cardiac Remodeling: Complement Activation and M2 Macrophages

**FIGURE 3:**
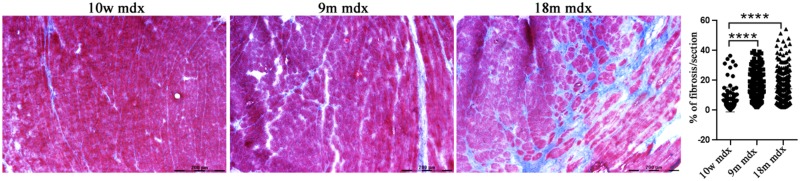
Evaluation of fibrosis in cardiac tissues of mdx mice at different ages. Representative Azan Mallory images of cardiac tissues of 10 weeks (10w), 9 months (9m), and 18m mdx mice (*n* = 5 each). Histogram represents the percentage of fibrotic area per cardiac section of mdx mice. Scale bar: 200 mm. One-way ANOVA with Tukey’s multiple comparisons test: ****p < 0.0001.

**FIGURE 4:**
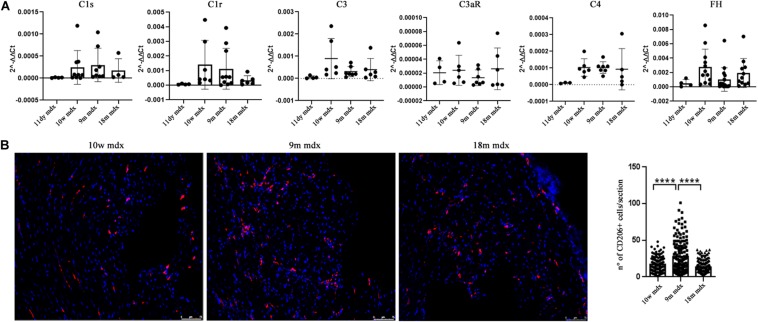
RT-qPCR of complement and evaluation of CD206 + cells in cardiac tissues of mdx mice at different ages. **(A)** RT-qPCR expression of genes involved in complement activation pathway in 11 days (11dy), 10 weeks (10w), 9 months (9m), and 18m mdx mice. **(B)** Immunofluorescence analysis of CD206 (red) in cardiac tissues of 10w, 9m, and 18m mdx mice (*n* = 5 each). Nuclei were counterstained with 4’,6-diamidino-2-phenylindole (DAPI) (blue) Scale bar: 75 mm. One-way ANOVA with Tukey’s multiple comparisons test: ****p < 0.0001. Each experiment was performed in triplicate wells. All values are expressed as the mean ± SD.

Considering the established involvement of PTX3 in fibrotic and inflammatory pathways, we aimed at discovering the effects of PTX3 upregulation and of the associated proteins in regulating the rising of pathological signs in dystrophic cardiac tissues. Azan Mallory staining of cardiac muscles showed a marked upregulation of fibrosis (calculated as a percentage of fibrosis per single image) in older mdx hearts (9m mdx and 18m mdx) related to 10w mdx mice (with *p* < 0.0001 for both). In line with previous results published by Van Erp et al. (2010), we demonstrated that the 18m mdx mice did not display a significant upregulation of fibrosis compared to 9m mice (Figure 3). Regulation of complement activation by PTX3 seems to be involved in tissue damage through a complex scheme. In brief, PTX3 can bind the C1q protein to activate the classical component cascade, allowing the deposition of C3 and C4 (Kunes et al., 2012). Alternatively, PTX3 can bind to the Factor H (FH) and limit the activation of the alternative pathway of complement (Ristagno et al., 2019). In this sense, we verified whether the elevated expression of PTX3 correlated to dysfunction of the complement signaling cascade in older 18m mdx mice. However, we did not find any significant variation in the expression of several components of complement cascade (Figure 4A). We then considered the infiltration of M1 and M2 F4/80 + macrophages (Van Erp et al., 2010) into mdx cardiac muscles and its age-related augment. Since PTX3 can coordinate the functions of macrophages, and macrophages themselves represent a source of PTX3, we counted the anti-inflammatory CD206 + M2 macrophages, finding a significant increase only in 9m mdx mice compared to 10w and 18m (with *p* < 0.0001 for both), ruling out a correlation between M2 macrophages and PTX3 expression (Figure 4B).

### Pentraxin 3-Dependent Pro-Inflammatory and Fibrotic Signaling: Immunoproteasomes, Alarmins, and Autophagic Markers

**FIGURE 5:**
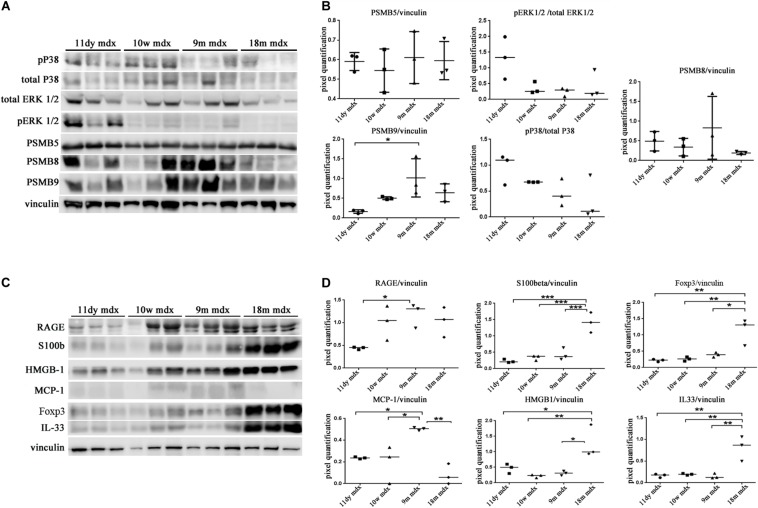
Western blot (WB) analysis of immunoproteasome (IP) subunits and alarmins in cardiac tissues of mdx mice at different ages. Representative WB of PSMB5, PSMB8, PSMB9, pERK/total ERK, and p38/total p38 in cardiac tissues of 11 days (11dy), 10 weeks (10w), 9 months (9m), and 18m mdx mice **(A)**. In the lateral panel, densitometric analysis of data, expressed as the ratio of different proteins versus vinculin in arbitrary units. One-way ANOVA with Tukey’s multiple comparisons test: *p < 0.05 **(B)**. Representative WB of receptor for advanced glycation end-products (RAGE), S100β, Foxp3, monocyte chemoattractant protein (MCP)-1, high-mobility group box (HMGB)1, and interleukin (IL)-33 in cardiac tissues of 11dy, 10w, 9m, and 18m mdx mice **(C)**. Data from densitometric analysis are expressed as the ratio of different proteins versus vinculin in arbitrary units in the lateral panel **(D)**. One-way ANOVA with Tukey’s multiple comparisons test: *p < 0.05; **p < 0.01; ***p < 0.001. Each experiment was performed in triplicate wells. All values are expressed as the mean ± SD.

**FIGURE 6:**
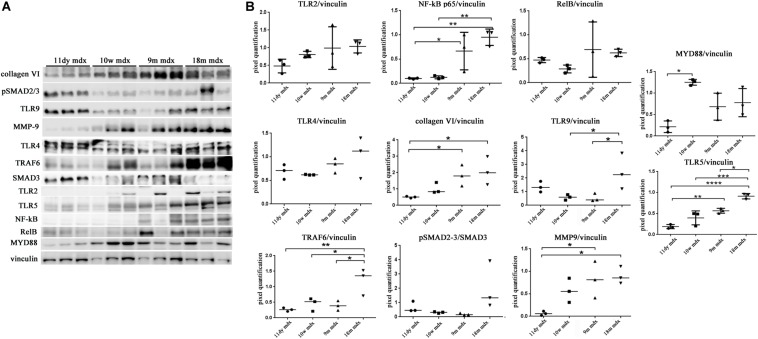
Evaluation of proteins involved in inflammation and fibrosis in cardiac tissues of mdx mice at different ages. Representative Western blot (WB) of several inflammatory and fibrotic mediators in cardiac tissues of 11 days (11dy), 10 weeks (10w), 9 months (9m), and 18m mdx mice **(A)**. Data from densitometric analysis are expressed as the ratio of different proteins versus vinculin in arbitrary units in the lateral panel. One-way ANOVA with Tukey’s multiple comparisons test: *p < 0.05; **p < 0.01; ***p < 0.001; ****p < 0.0001 **(B)**. Each experiment was performed in triplicate wells. All values are expressed as the mean ± SD.

We moved to investigate other pathways whose activity could foster PTX3 upregulation and modulate the inflammatory and dystrophic cues. Among these pathways, we considered PTX3 expression dependency on the IP and ERK1/2–p38MAPK activity, as already demonstrated in myocardial inflammation (Paeschke et al., 2016). However, in mdx mice, we did not see any difference except for a slightly higher expression of PSMB9 in 9m mice (9m mdx versus 11dy mdx, *p* = 0.0193) (Figures 5A,B). Therefore, we looked into alarmins that are recognized by specific receptors such as RAGE and TLRs: alarmins are activated through their ligand binding by different signaling cascades (as those dependent on IL-1R/MYD88 and MAPKs) leading to NF-κB and inflammatory cytokines release which might influence PTX3 expression and fibrosis development. In this case, we discovered that the amount of inflammatory and fibrotic proteins increases in mdx 18m cardiac muscles, likely explaining a more jeopardized pathological phenotype. Among alarmins, the S100β (18m mdx versus 9m mdx, *p* = 0.0009; versus 10w mdx, *p* = 0.0005; versus 11dy mdx, *p* = 0.0002), HMGB1 (18m mdx versus 9m mdx, *p* = 0.0447; versus 10w mdx, *p* = 0.0047; versus 11dy mdx, *p* = 0.0017), and IL-33 (18m mdx versus 9m mdx, *p* = 0.0027; versus 10w mdx, *p* = 0.0042; versus 11dy mdx, *p* = 0.0031) have been found significantly increased according to the age. Instead, RAGE expression was not dependent on the age, but its expression was elevated in mdx 9m hearts related to 11dy mdx mice (*p* = 0.0494). Furthermore, we envisaged a role of Treg cells in the dystrophic framework, in line with previous literature describing how the phenotype and the specialization of Tregs were determined by IL-33/ST2 (Pastille et al., 2019) and IL-1/IL-33 (Alvarez et al., 2019) pathway, respectively. Since it is known that Tregs are abundant in mdx necrotic muscles (Burzyn et al., 2013), we were not surprised to find that Foxp3 amount was more abundant in older mdx mice compared to the younger (18m mdx versus 9m mdx, *p* = 0.0107; 18m mdx versus 10w mdx, *p* = 0.0041; 18m mdx versus 11dy mdx, *p* = 0.0031). MCP-1 protein, involved in fibrosis and inflammatory events, was also upregulated in mdx mice at 9 months related to the other ages (9m mdx versus 18m mdx, *p* = 0.0035; 9m mdx versus 10w mdx, *p* = 0.0205; 9m mdx versus 11dy mdx, *p* = 0.0433) (Figures 5C,D). It was described that cardiac HMGB1 overexpression can mediate a pro-inflammatory immune response independently from RAGE (Bangert et al., 2016). Accordingly, in mdx cardiac muscles, we demonstrated an upregulation of other alarmin receptor expression, according to the age: TLR4 and TLR5 (18m mdx versus 9m mdx, *p* = 0.0101; 18m mdx versus 10w mdx, *p* = 0.0008; 18m mdx versus 11dy mdx, *p* < 0.0001; 9m mdx versus 11dy mdx, *p* = 0.0062) and TLR9 (18m mdx versus 9m mdx, *p* = 0.0465; 18m mdx versus 10w mdx, *p* = 0.0470). This condition further promoted the overexpression of NF-κB-p65 (18m mdx versus 10w mdx, *p* = 0.0060; 18m mdx versus 11dy mdx, *p* = 0.0051; 9m mdx versus 11dy mdx, *p* = 0.0457) and TRAF6 (18m mdx versus 9m mdx, *p* = 0.0198; 18m mdx versus 10w mdx, *p* = 0.0287; 18m mdx versus 11dy mdx, *p* = 0.0088) in older mdx mice. In turn, alarmins/TLRs/TRAF6-NF-κB pathway allowed the overexpression of PTX3 and the promotion of fibrosis, as highlighted by the upregulation of collagen VI (18m mdx versus 11dy mdx, *p* = 0.0367; 9m mdx versus 11dy mdx, *p* = 0.0418) and MMP9 (18m mdx versus 11dy mdx, *p* = 0.0192; 9m mdx versus 11dy mdx, *p* = 0.0331) (Figures 6A,B).

**FIGURE 7:**
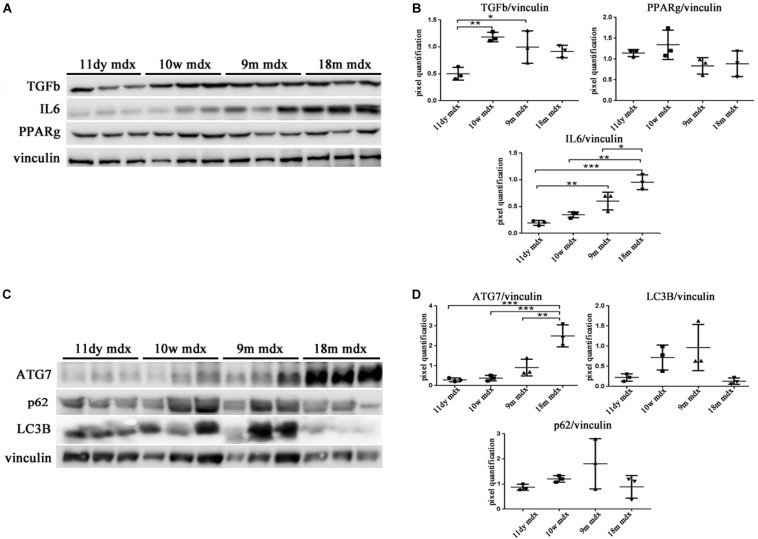
Evaluation of inflammatory cytokines and autophagic mediators in cardiac tissues of mdx mice at different ages. Representative Western blot (WB) of transforming growth factor (TGF)-b, poly(ADP-ribose) polymerase (PPAR)g, interleukin (IL)-6, and tumor necrosis factor (TNF)-a in cardiac tissues of 11 days (11dy), 10 weeks (10w), 9 months (9m), and 18m mdx mice **(A)**. Data from densitometric analysis are expressed as the ratio of different proteins versus vinculin in arbitrary units in the lateral panel. One-way ANOVA with Tukey’s multiple comparisons test: *p < 0.05; **p < 0.01; ***p < 0.001 **(B)**. Representative WB of ATG-7, LC3B, and p62 in cardiac tissues of 11dy, 10w, 9m, and 18m mdx mice **(C)**. Data from densitometric analysis are expressed as the ratio of different proteins versus vinculin in arbitrary units in the lateral panels. One-way ANOVA with Tukey’s multiple comparisons test: **p < 0.01; ***p < 0.001 **(D)**. Each experiment was performed in triplicate wells. All values are expressed as the mean ± SD.

Within this environment, we determined the amount of IL-6 pro-inflammatory cytokine, finding a significant augment in older compared to younger mdx mice (18m mdx versus 9m mdx, *p* = 0.0225; 18m mdx versus 10w mdx, *p* = 0.0008; 18m mdx versus 11dy mdx, *p* = 0.0002; 9m mdx versus 11dy mdx, *p* = 0.0100). Moreover, we established that even though the TGF-β extent was higher in 10w mdx mice and lower in 9m mdx related to younger animals (9m mdx versus 11dy mdx, *p* = 0.0366; 10w mdx versus 11dy mdx, *p* = 0.0066), its expression did not vary significantly with the age, as described in Van Erp et al. (2010) (Figures 7A,B). PTX3 has been shown to regulate mitochondrial membrane potential and apoptosis (Lee et al., 2018) and, *vice versa*, its expression can be controlled by ATG7 (Qiang et al., 2017). Thus, we investigated the expression of autophagy markers ATG7, p62, and LC3B, and we found an increased ATG7 expression at 18 months of age (18m mdx versus 9m mdx, *p* = 0.0026; 18m mdx versus 10w mdx, *p* = 0.0004; 18m mdx versus 11dy mdx, *p* = 0.0003), confirming a dependency on the age (Figures 7C,D). Following these results, we performed a statistical test to determine whether there was a significant correlation between the expression of PTX3 and the proteins whose amount was significantly modified in mdx cardiac tissues at different ages. Interestingly, we found that there was a positive significant correlation dependent on the age between PTX3 and inflammatory and fibrotic players such as S100β and HMGB1, TLR5, IL-6, MMP9, and TRAF6 (Table 2A). Thus, the expression of other proteins (Foxp3, TRAF6, ATG7) was correlated with PTX3 independently from the age (Table 2B). In addition, we performed a multiple regression analysis, and we obtained a model of PTX3 considering S100β, HMGB1, TLR5, and TLR9 (corrected *r*^2^ = 0.91), suggesting their involvement in a common pathway.

**TABLE 2:** Statistical analysis of pentraxin (PTX)3 expression.

Correlation Ptx3	r	*P*-value
Age	0.898^#^	<0.0001
MMP9	0.571^#^	0.0524
HMGB1	0.866^#^	0.0003
S100β	0.959^#^	<0.0001
IL-6	0.871^#^	0.0002
NF-κB	0.727^#^	0.0074
TLR5	0.871^#^	0.0002
Foxp3	0.825*	0.001
IL-33	0.545*	0.0666
TRAF6	0.797*	0.0019
ATG7	0.839*	0.0006
TLR9	0.154*	0.6331

r Pearson, r Spearman, and p-value confirmed the positive correlation between the expression of PTX3 and high-mobility group box (HMGB)1, S100β, and Foxp3 (**A**); Toll-like receptor (TLR)5 and TRAF-6 (**B**); autophagy-related (ATG)-7 (**C**). ^#^ r Pearson. * r Spearman. The bold values are statistically significant according to the value of p.

### ONX-0914 Modulates Pentraxin 3 Expression in Cardiac Tissues of 9m Mdx Mice

**FIGURE 8:**
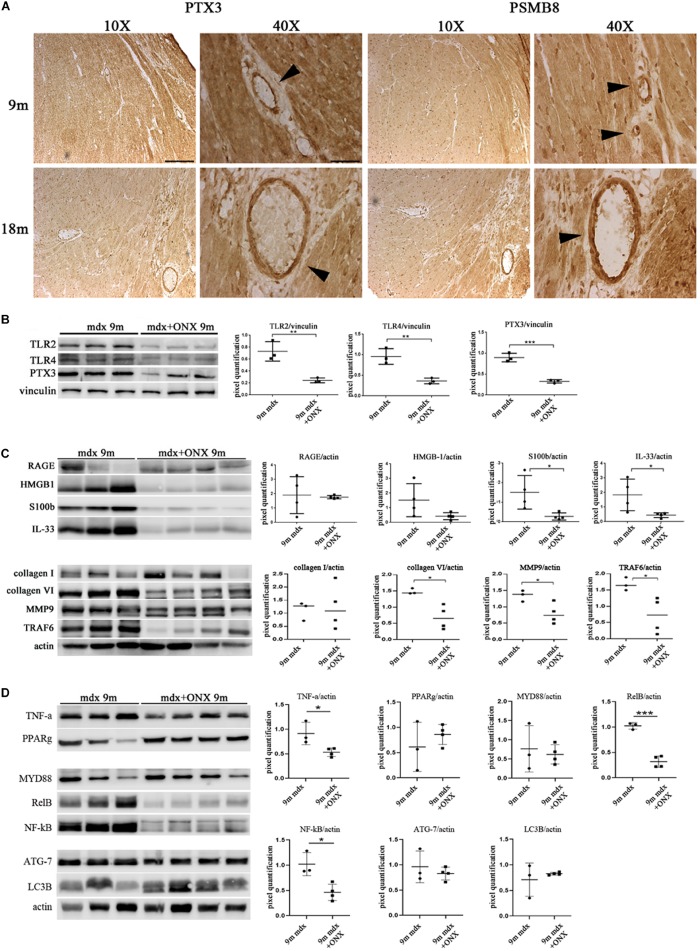
Modulation of proteins in cardiac tissues of mdx mice at different ages following ONX-0914 treatment. Representative images of pentraxin (PTX)3 and PMSB8 immunohistochemistry staining of cardiac tissues showing PTX3- and PSMB8-positive endothelial cells (arrowheads) in cardiac tissues from 9 months (9m) and 18m mdx mice. Image magnifications: 10 × (scale bar: 200 mm) and 40 × (scale bar: 50 mm) **(A)**. Representative Western blot (WB) in cardiac tissues of untreated and ONX-0914-treated 9m mdx mice for Toll-like receptor (TLR)2, TLR4, PTX3 **(B)**; receptor for advanced glycation end-products (RAGE), high-mobility group box (HMGB)1, S100β, and interleukin (IL)-33; collagen I, collagen VI, matrix metalloproteinase (MMP)9, and TRAF-6 **(C)**; tumor necrosis factor (TNF)-a, poly(ADP-ribose) polymerase (PPAR)g, MYD88, RelB, nuclear factor (NF)-κB, ATG-7, and LC3B **(D)**. Data from densitometric analysis are expressed as the ratio of different proteins versus vinculin **(B)** and actin **(C,D)** in arbitrary units in the lateral panels. Student’s *t*-test: **p < 0.01; ***p < 0.001 **(B)**; *p < 0.05 **(C)**; *p < 0.05; ***p < 0.001 **(D)**. Each experiment was performed in triplicate wells. All values are expressed as the mean ± SD.

We have determined that PTX3 could be an important target to modulate the inflammatory/fibrotic pathways in dystrophic cardiac tissues. Upregulation of PTX3 in cardiomyocytes is dependent on IP, whose inhibition has been shown to be effective in downregulating PTX3 expression and other NF-κB-dependent pathways (Voigt, personal communication). We have also demonstrated that dystrophic murine hearts treated with IP inhibitor ONX-0914 witness a downregulation of the expression of fibrotic mediators such as STAT3, STAT1, OPN, and ERK1/2 (Farini et al., 2019). We firstly confirmed the occurrence and the increase of IP subunits PSMB8 and PSMB9 in mdx hearts of different ages (Figure 5A). Interestingly, both PTX3 and PSMB8 signals were clearly detectable by immunohistochemistry in tight correlation with endothelial cells of cardiac vessels in 9m and 18m mdx mice (Figure 8A). To strengthen the possibility that IP played a role in controlling inflammatory/fibrotic pathways leading to PTX3 expression, a selective inhibition of the IP subunit PSMB8 by ONX-0914 supplementation was performed on mdx mice. In the cardiac tissues of 10w mdx mice, we demonstrated that the ONX-0914 did not vary significantly the expression of autophagic, fibrotic, and inflammatory markers (Supplementary Figure S1). In older mdx mice, we found instead that there was a significant downregulation of PTX3 (*p* = 0.0008) and TLR2 (*p* = 0.0074) and TLR4 (*p* = 0.0072), whose activities are fundamental for PTX3 activation (Figure 8B). We confirmed that ONX-0914 treatment was effective in reducing significantly the amount of IL-33 (*p* = 0.0441) and alarmins HMGB1 and S100β (*p* = 0.0306), while the expression of RAGE did not vary (Figure 8C). Furthermore, in line with our previous work (Farini et al., 2019), we found that different proteins involved in fibrotic development—whose activity could be dependent on PTX3 expression – were downregulated in ONX-0914-treated mice (collagen VI, *p* = 0.0148; MMP9, *p* = 0.0400; TRAF6, *p* = 0.0377) (Figure 8C). Importantly, we determined a significant downregulation of key mediators of fibrosis as TNF-a (*p* = 0.0258), RelB (*p* = 0.0002), and NF-κB (*p* = 0.0123), whose importance in PTX3 modulation had been previously demonstrated (Li et al., 2020; Figure 8D). However, the expression of autophagy markers such as ATG7 and LC3B was not modified by ONX-0914 treatment (Figure 8D).

### Network-Based Prediction of Cardiac Tissues of Mdx Recapitulates the Pentraxin 3 Protein Interactions Involving Inflammatory/Fibrotic Pathways

**FIGURE 9:**
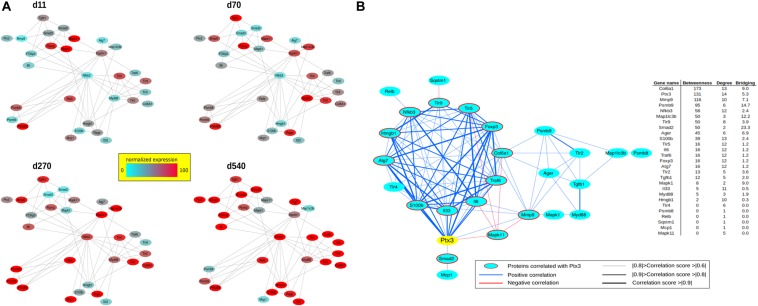
Protein–protein interaction (PPI) and co-expression network of proteins differentially expressed at days (d)11, d70, d270, and d540. PPIs were retrieved by STRING Cytoscape’s App; only Experimental and Database annotated interactions, with a score > 0.15, were considered. Protein expression normalized in range 0–100 (%) **(A)**. Betweenness, Degree, and Bridging centralities calculated by Centiscape Cytoscape’s App. In red and bold centrality values above the average calculated on whole network; nodes with Betweenness, Degree, and Bridging above the average were considered hubs (genes in red) **(B)**.

**FIGURE 10:**
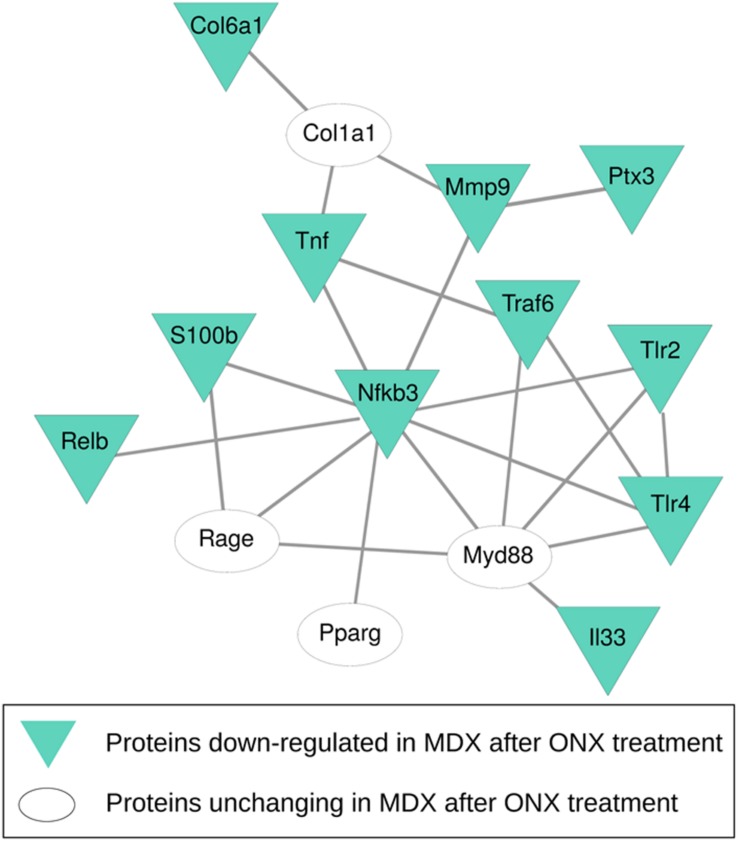
Protein–protein interaction (PPI) network of proteins differentially expressed after ONX-0914 treatment. PPIs were retrieved by STRING Cytoscape’s App; only Experimental and Database annotated interactions, with a score > 0.15, were considered. Blue triangles indicate proteins downregulated after ONX-0914 treatment.

To investigate the correlation of PTX3 expression with inflammatory/fibrotic pathways of mdx cardiac tissues, we reconstructed the PPIs retrieved from bioinformatic STRING analysis of the differentially expressed proteins of cardiac tissues in mdx mice at different ages (11, 70, 270, and 540 days old). In these analyses, we counted 30 nodes and 70 edges. During aging, inflammatory mediators, such as alarmins, NF-κB, RAGE, TLRs, and RelB, increased earlier and progressively peaked in cardiac tissues of 540 days old mdx (Figure 9A). In addition to these inflammatory proteins, we found cardiac increase of IP subunits throughout the life of the mdx mice (Figure 9A). On the other hand, the fibrotic remodeling of mdx hearts begins from 70 days old and peaks at 540 days old according to the increase of TGFβ1, MMP9, and collagen VI proteins (Figure 9A). The critical role of PTX3 in promoting the inflammatory pathway was confirmed by its early expression in cardiac tissues of 11dy mdx mice. Moreover, cardiac PTX3 upregulation was observed after activation of TLR4/MMP9 pathway in 270 and 540 days old mdx (Figure 9A). The results collectively suggested that PTX3 and IP might be both the targets of similar inflammatory/fibrotic pathways. By evaluating the structure of PPI networks, Nfkb3 (STAT3) could be considered as a protein hub due to its number of interactions as well as its central role in connecting different clusters of nodes (Figures 9, 10). Similarly, PTX3, collagen VI (Col61a1), MMP9, and PSMB9 resulted hubs in the protein co-expression network showing centrality values above the average calculated on the whole network (Figure 9B). Moreover, PTX3 resulted in the most correlated protein in the observed inflammatory/fibrotic pathway (Degree = 14) (Figure 9B) with higher correlation (score > 0.9) to Foxp3, ATG7, and S100β (Supplementary Figure S1). Most PTX3 correlations resulted positive, while a single negative correlation was found between PTX3 and MAPK11 (Supplementary Figure S1). Besides, at 270 days in age, the mdx heart is affected by a cardiomyopathy (Quinlan et al., 2004, p. 491). Especially striking was the decrease of PTX3 expression and inflammatory/fibrotic pathway downregulation following treatment with ONX-0914 in hearts of 270 days old mdx (Figure 10).

## Discussion

Pentraxins are key components of the humoral innate immune system: the long pentraxin PTX3 is highly expressed during tissue-damaging events and, in turn, coordinates inflammation and vascular remodeling (Manfredi et al., 2008). Despite a recent interest in the measurement of pentraxins in ischemic heart disorders (Peri et al., 2000; Salio et al., 2008; Paeschke et al., 2016), there is no evidence as to their actual role in the pathogenesis of dystrophic cardiomyopathy. In this study, we demonstrated that dystrophic cardiac expression of PTX3 correlated positively with age and inflammatory/fibrotic pathways. Expression of PTX3 in the heart had already been observed at later stages of inflammation (Nebuloni et al., 2011). Accordingly, Pucci et al. (2014) demonstrated the fundamental role of M2 macrophages in determining the higher amount of PTX3 in arteriosclerotic plaque development. Moreover, PTX3 can interact with endothelial cells and, through the binding with P-selectin, can modulate the recruitment of monocytes and macrophages at the inflammatory sites (Deban et al., 2010). Interestingly, the IP modulates PTX3 expression through a complex mechanism involving TLRs, NF-κB, MAP kinases (Paeschke et al., 2016). According to these premises, we found co-expression of the IP subunit PSMB8 and PTX3 in mdx hearts and reduced expression of PTX3 after IP inhibition. Thus, local dystrophic cardiac expression of PTX3 might extend the well-known role of circulating PTX3 as a biomarker (Salio et al., 2008). In fact, we found aging-associated upregulation of PTX3 in muscles and hearts of mdx mice with predominant localization in fibrotic areas and, more interestingly, in vessels and perivascular areas. Interestingly, we found no correlation between PTX3 expression and M2 macrophages of mdx hearts, suggesting a specific role of dystrophic endothelial cells in the release or expression of PTX3. These evidences are in accordance with data provided from several labs describing PTX3 as a biomarker of endothelial dysfunctions, regulating nitric oxide, P-selectin, and fibroblast growth factor-2 production (Kotooka et al., 2008; Yasunaga et al., 2014; Carrizzo et al., 2015). Strong evidences suggest that complement components amplify tissue damages recruiting leukocytes: in the present study, we found that PTX3 expression was not influenced by complement components; conversely, alarmins facilitate inflammatory reactions and consequent fibrosis participating to the PTX3 secretion (Tsoporis et al., 2012; Bangert et al., 2016). Among alarmins’ receptors, the TLR5 is commonly expressed in cardiomyocytes and endothelial cells (Hayashi et al., 2001) and enhances cardiac innate immune responses (Rolli et al., 2010). Recently, it was shown that TLR5 inhibition ameliorates cardiac fibrosis by modulating inflammation and tissues’ remodeling (Liu et al., 2015). Other studies determined that HMGB1 blockade alleviates myocardial fibrosis (Wang et al., 2014), possibly interfering with the TLR2-HMGB1 ligand and cardiac autophagy (Wu et al., 2018; Liu et al., 2019). Consistent with these evidences, we demonstrated that mdx cardiac dysfunctions could be partly due to an inflammatory signaling pathway in which PTX3 is involved together with S100β, HMGB1, TLR5, and TLR9, with consequent increment of collagen deposition and fibrosis. Since PTX3 has been shown to regulate apoptosis (Lee et al., 2018) and its expression can be controlled by ATG7 (Qiang et al., 2017), we investigated the expression of autophagy markers ATG7, p62, and LC3B, and we found an increased ATG7 expression in older cardiac mdx. However, expression of PTX3 in cardiomyocytes is dependent on IP activity (Paeschke et al., 2016), and we have already demonstrated that IP inhibitor ONX-0914 had a fundamental role in modulating the pathological phenotype in skeletal (Farini et al., 2016) and cardiac (Farini et al., 2019) muscles of mdx mice. Since we found that PTX3 and IP were co-expressed in the fibrotic/inflammatory areas in cardiac tissues, we explored the effects of ONX-0914 in mediating PTX3-dependent pathways, confirming a downregulation of expression levels of alarmins-, collagen-, and NF-κB-dependent proteins. While critical for antigen presentation, the IP of endothelial cells may be a key link between inflammatory factors and vascular cell remodeling and thus may be an important factor in myocardial damage of mdx, as previously described in myocardial infarction (Yang et al., 2009). Interestingly, we noted PTX3 and PSMB8 co-expression in dystrophic mdx cardiac vessels. Moreover, we found that myocardial damage of older mdx triggered expansion of NG2 + pericyte population with altered morphology. In adult mouse hearts, endothelial cells and pericytes are the most abundant non-cardiac muscle cells (Nees et al., 2012; Pinto et al., 2016). Considering their abundance, phenotypic plasticity, and functional diversity, endothelial cells and pericytes may be critically involved in regulating inflammatory, fibrotic, angiogenic, and reparative responses in dystrophic hearts of mdx mice. Meanwhile, the network-based prediction analysis of changed inflammatory/fibrotic proteins raised the possibility of Nfkb3 (STAT3) as an important hub node. Importantly, endothelial cell-released STAT3 has a key role in inflammation that underlies cardiovascular disease, and conversely, cardiomyocyte STAT3 is important for maintaining endothelial cell functions and the capillary integrity (Zouein et al., 2019). Furthermore, PTX3 resulted the most correlated protein in the inflammatory/fibrotic pathway with higher correlation to Foxp3, ATG7, and S100β proteins that have been linked to endothelial cell functions (Kreisel et al., 2004; Krupnick et al., 2005; Pober and Sessa, 2007; Tsoporis et al., 2010; Singh et al., 2015; Vion et al., 2017). Overall, these results provide the first stringent correlation between PTX3 cardiac expression and inflammatory/fibrotic pathways in an animal model of DMD. So far, data available propose a role for PTX3 as a predictive marker of fibrosis in dystrophic cardiac tissues. In general, PTX3 levels in dystrophic cardiac tissues rise first, reflecting the inflammatory response affecting myocardial damage and subsequently modulating the fibrotic response. However, PTX3 may have different kinetics of production and different patterns of recognized ligands between inflammatory and fibrotic pathways. On this regard, high PTX3 levels were associated with dystrophic cardiac vessels. The evidence for a regulatory role in the pathogenesis of dystrophic cardiomyopathy provides further incentive to the assessment of the clinical relevance of PTX3 measurement in prognostic value and in guiding therapy for cardiomyopathy of DMD.

## Data Availability Statement

All datasets generated for this study are included in the article/Supplementary Material.

## Ethics Statement

The animal study was reviewed and approved by all procedures involving living animals were performed in accordance with Italian law (D.L.vo 116/92 and subsequent additions), which conforms to the European Union guidelines. The use of animals in this study was authorized by the National Ministry of Health (protocol number 10/13–2014/2015).

## Author Contributions

YT, AF, CS, and PM conceived and designed the experiments. AF, YT, and CV wrote the manuscript. PB, LT, CS, DD, and RR interpreted and analyzed the data. AF, PB, LT, CV, and SG performed the experiments and acquired the data. All authors stated were involved in the critical revision of the manuscript and approved the final version of the manuscript, including the authorship list. YT had full access to all the data in the study and had final responsibility for the decision to submit for publication.

## Conflict of Interest

The authors declare that the research was conducted in the absence of any commercial or financial relationships that could be construed as a potential conflict of interest.

## Footnotes

1 https://string-db.org/

2 http://rsbweb.nih.gov/ij/

## Supplementary Material

FIGURE S1 PTX3 correlation network. Proteins correlated with PTX3 and their expression values at day (d)11, d70, d270, and d540. Protein expression was normalized in range 0–100 (%).
