# Supplementary figures and images for: PTX3 Predicts Myocardial Damage and Fibrosis in Duchenne Muscular Dystrophy
Source: Front Physiol. 2020 May 19;11:403. doi: 10.3389/fphys.2020.00403 (PMC7248204; doi:10.3389/fphys.2020.00403)

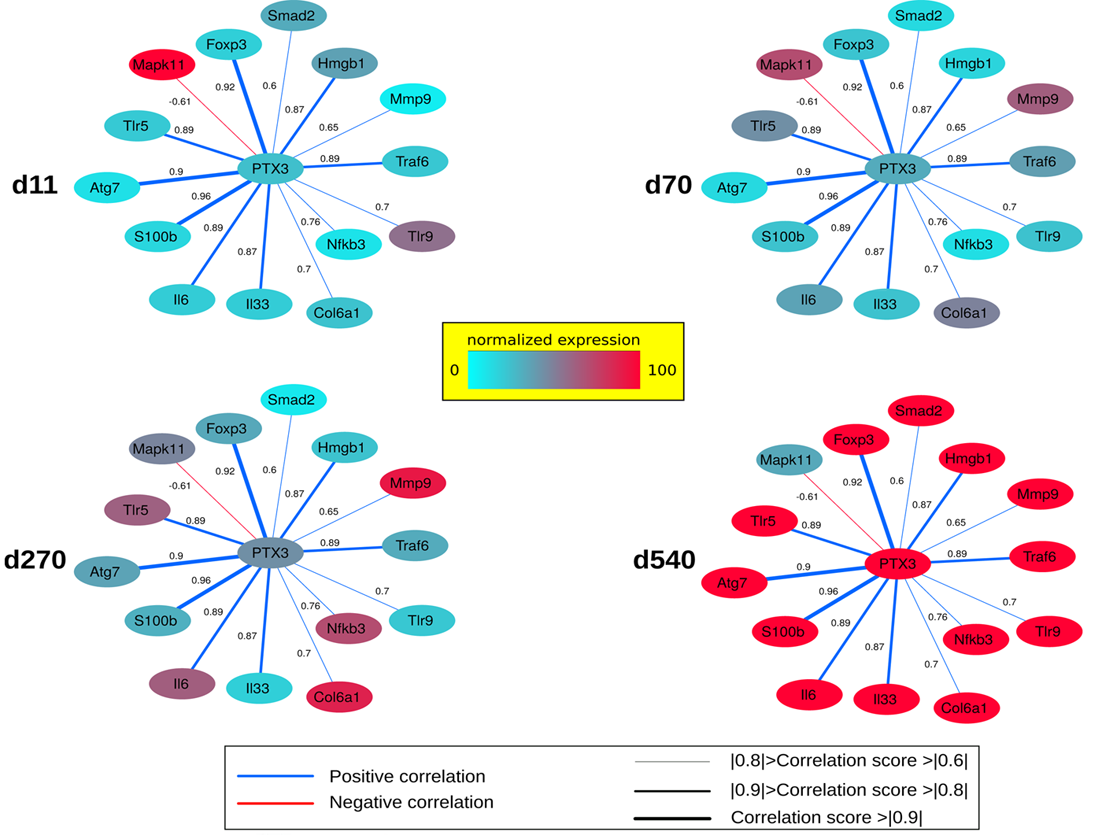

Supplement: FIGURE S1 — PTX3 correlation network. Proteins correlated with PTX3 and their expression values at day (d)11, d70, d270, and d540. Protein expression was normalized in range 0–100 (%). [file Image_1.TIF]
